# Supplementary figures and images for: The mechanosensitive ion channel PIEZO1 promotes satellite cell function in muscle regeneration
Source: Life Sci Alliance. 2022 Nov 29;6(2):e202201783. doi: 10.26508/lsa.202201783 (PMC9711862; doi:10.26508/lsa.202201783)

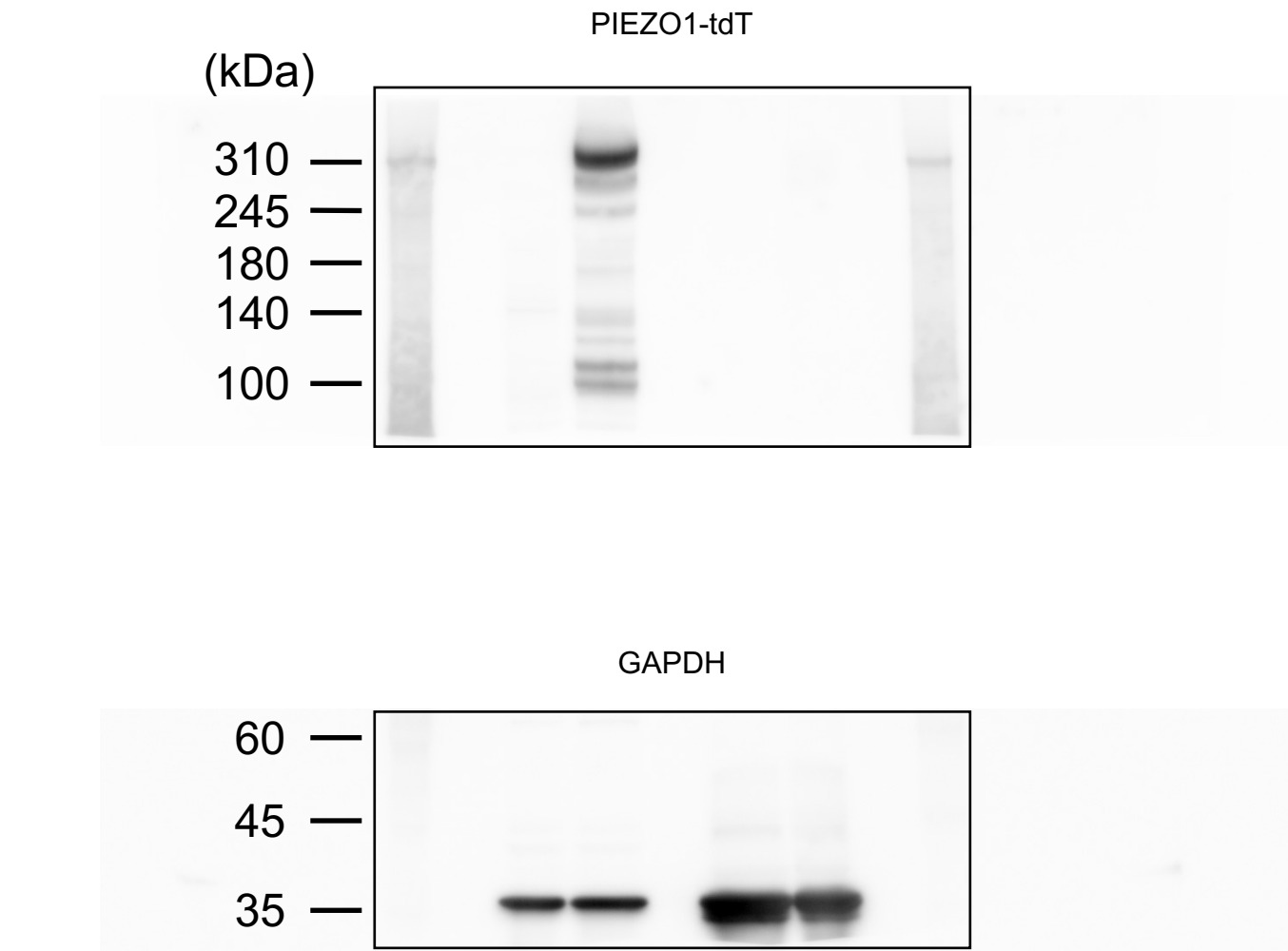

Supplement: Supplementary file 1 [file LSA-2022-01783_SdataF1.pdf]
